# Parallel descending dopaminergic connectivity of A13 cells to the brainstem locomotor centers

**DOI:** 10.1038/s41598-018-25908-5

**Published:** 2018-05-22

**Authors:** Sandeep Sharma, Linda H. Kim, Kyle A. Mayr, David A. Elliott, Patrick J. Whelan

**Affiliations:** 1 Hotchkiss Brain Institute, University of Calgary, Calgary, AB T2N 4N1 Canada; 2 Department of Comparative Biology and Experimental Medicine, University of Calgary, Calgary, AB T2N 4N1 Canada; 3 Hotchkiss Brain Institute Advanced Microscopy Platform, University of Calgary, Calgary, AB T2N 4N1 Canada

## Abstract

The mesencephalic locomotor region (MLR) is an important integrative area for the initiation and modulation of locomotion. Recently it has been realized that dopamine (DA) projections from the substantia nigra pars compacta project to the MLR. Here we explore DA projections from an area of the medial zona incerta (ZI) known for its role in motor control onto the MLR. We provide evidence that dopaminergic (DAergic) A13 neurons have connectivity to the cuneiform nucleus (CnF) and pedunculopontine tegmental nucleus (PPTg) of the MLR. No ascending connectivity to the dorsolateral striatum was observed. On the other hand, DAergic A13 projections to the medullary reticular formation (MRF) and the lumbar spinal cord were sparse. A small number of non-DAergic neurons within the medial ZI projected to the lumbar spinal cord. We then characterized the DA A13 cells and report that these cells differ from canonical DA neurons since they lack the Dopamine Transporter (DAT). The lack of DAT expression, and possibly the lack of a dopamine reuptake mechanism, points to a longer time of action compared to typical dopamine neurons. Collectively our data suggest a parallel descending DAergic pathway from the A13 neurons of the medial ZI to the MLR, which we expect is important for modulating movement.

## Introduction

Dopaminergic (DAergic) locomotor control is associated with indirect innervations via ascending projections from the substantia nigra pars compacta (SNc) to the dorsal striatum (DSTR) that project onto the brainstem locomotor regions, and the motor cortex. The mesencephalic locomotor region (MLR) is a crucial brainstem region that controls locomotion in vertebrates^1–4^. The pedunculopontine tegmental nucleus (PPTg) and the cuneiform nucleus (CnF) are considered part of the MLR in mammals^5^. The presence of DAergic fibers in MLR have been reported in lamprey^6^, salamander^7^, rat^7,8^, monkey^9^ and human^10^ suggesting an evolutionarily conserved DAergic innervation of the MLR^11^. Ryczko and colleagues^7^ discovered an exclusive descending DAergic projection from SNc to PPTg of the MLR separate from the classic ascending DAergic nigrostriatal circuitry. The stimulation of the diencephalic DAergic region called posterior tuberculum, an area homologous to the mammalian SNc as well as ventral tegmental area (VTA)^12,13^, releases dopamine (DA) in the MLR of lampreys^6^ and salamanders^7^. Furthermore, in rats^7^, SNc stimulation resulted in DA release in the MLR suggesting this parallel descending DAergic pathway is conserved and functional in mammals.

This recent discovery of parallel DAergic SNc projections to the MLR raises the possibility of such projections from other DAergic cell groups. Ryczko *et al*.^7^ reported that the DAergic A11 neurons known to send descending projections to the spinal cord^14,15^ do not project to the PPTg nucleus. DAergic A13 cells occupy a region in the medial aspect of the ZI, which in turn projects to a diverse range of targets that are important in motor control including the cerebellum, spinal cord, and red nucleus^16^. However, it remains to be tested if DAergic A13 cells project to the MLR. Indeed, electrical and chemical stimulation of the medial zona incerta (ZI) in diencephalon is reported to affect locomotion and posture in cats^2,17–19^ and rats^20–22^. The main objective of this study is to explore the descending connectivity of DAergic A13 cells to the CnF and PPTg of the MLR using retrograde tracer fluorogold (FG) injections and immunostaining for tyrosine hydroxylase (TH). We also test the possibility of ascending DAergic A13 projections to the DSTR, a major input structure for DA within the nigrostriatal circuitry. To better define the descending DAergic connectome we examine projections to the medullary reticular formation (MRF), a well-known source of reticulospinal (RS) cells, and a hub for control of spinal locomotor circuits^23^. The detailed description of anatomical and functional MRF has been discussed elsewhere (reviews^23–25^). In rodents, much effort has focused on gigantocellular reticular nucleus (Gi) and magnocellular nucleus of medulla which encompasses the lateral paragigantocellular nucleus (LPGi), gigantocellular reticular nucleus alpha (GiA) and ventral section (GiV), respectively^24–27^. These nuclei form the reticulospinal pathway and contain cells that descend ipsilaterally via the ventrolateral and ventromedial funiculi^28,29^. However, there is a lack of information on descending DAergic A13 connectivity to MRF and there are conflicting reports on DAergic A13 projections to the spinal cord^14^. Therefore, we examined descending DAergic projections to the Gi region of MRF and the lumbar spinal cord in addition to the MLR. Our work demonstrates that the DAergic A13 nucleus provides a parallel descending dopaminergic connectivity to the MLR but not the ascending DAergic connectivity to DSTR. These data expand our understanding of the descending DAergic connectome onto locomotor regions of the brainstem, providing evidence of direct DAergic pathways from the medial ZI onto the MLR. A portion of these data were published in an abstract form^30^.

## Results

### Distribution and quantification of double-labeled TH-ir/FG^+^ neurons in A13

#### Cuneiform nucleus

**Figure 1:**
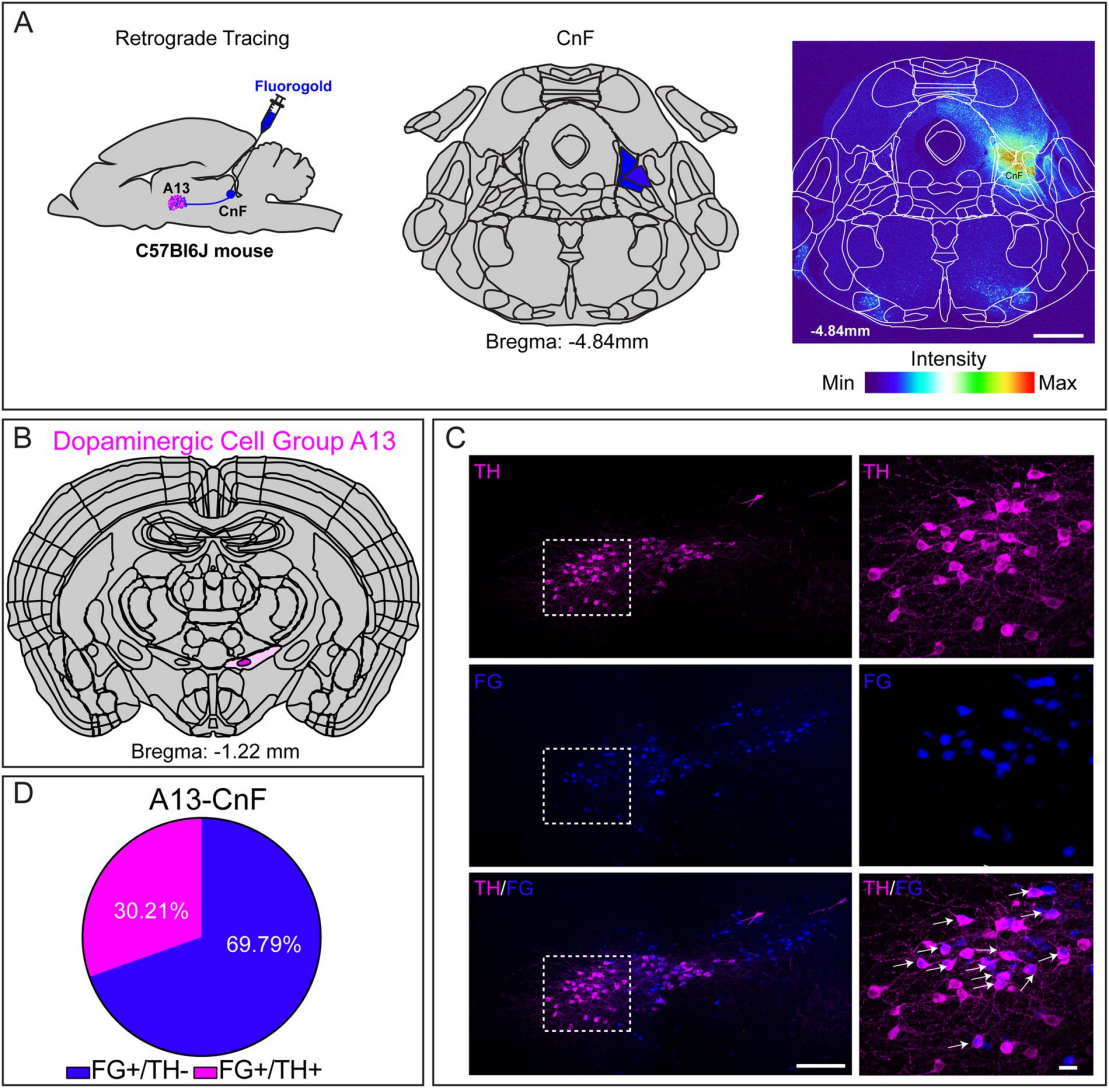
Dopaminergic A13 cells project to the CnF. (**A**–**D**) Many of the FG labelled cells (30.2%; n = 6 mice) co-expressed TH-ir. Scale bars A = 1000 µm, B = 100 µm and 20 µm. Atlas images adapted from template available from Allen Mouse Brain Atlas (2004)^39,90,91^.

To establish connectivity between DAergic A13 neurons and CnF in the dorsal MLR, we injected a retrograde tracer Fluoro-Gold (FG) into the CnF region (Fig. 1A). The core of FG injection and the spread was quantified in both the rostral and caudal directions from the injection site (Bregma: −4.04 mm to −5.20 mm). The animals included in the analysis had CnF labeled as the core of the injection site (Supplementary Figure 1). Spread from the core region abutted neighbouring areas including external nucleus of inferior colliculus (ECIC), lateral lemniscus (LL), midbrain reticular nucleus (MRN), motor related deep grey layer of superior colliculus (SCdg), ventral lateral periaqueductal grey (vlPAG) and the dorsal roof of PPTg. We observed dense FG positive (FG^+^) cell bodies in A13 (227.8 ± 24.68 cells; n = 6 mice; Fig. 1B,C) in addition to the A11, ZI and LH (data not shown). We observed that 30.21% of total FG^+^ cells (68.83 ± 9.33 cells; n = 6 mice; Fig. 1D) of A13 were TH immunoreactive (TH-ir). These results suggest DAergic A13 neurons provide a key contribution in terms of DAergic innervation of the CnF.

#### Pedunculopontine tegmental nucleus

**Figure 2:**
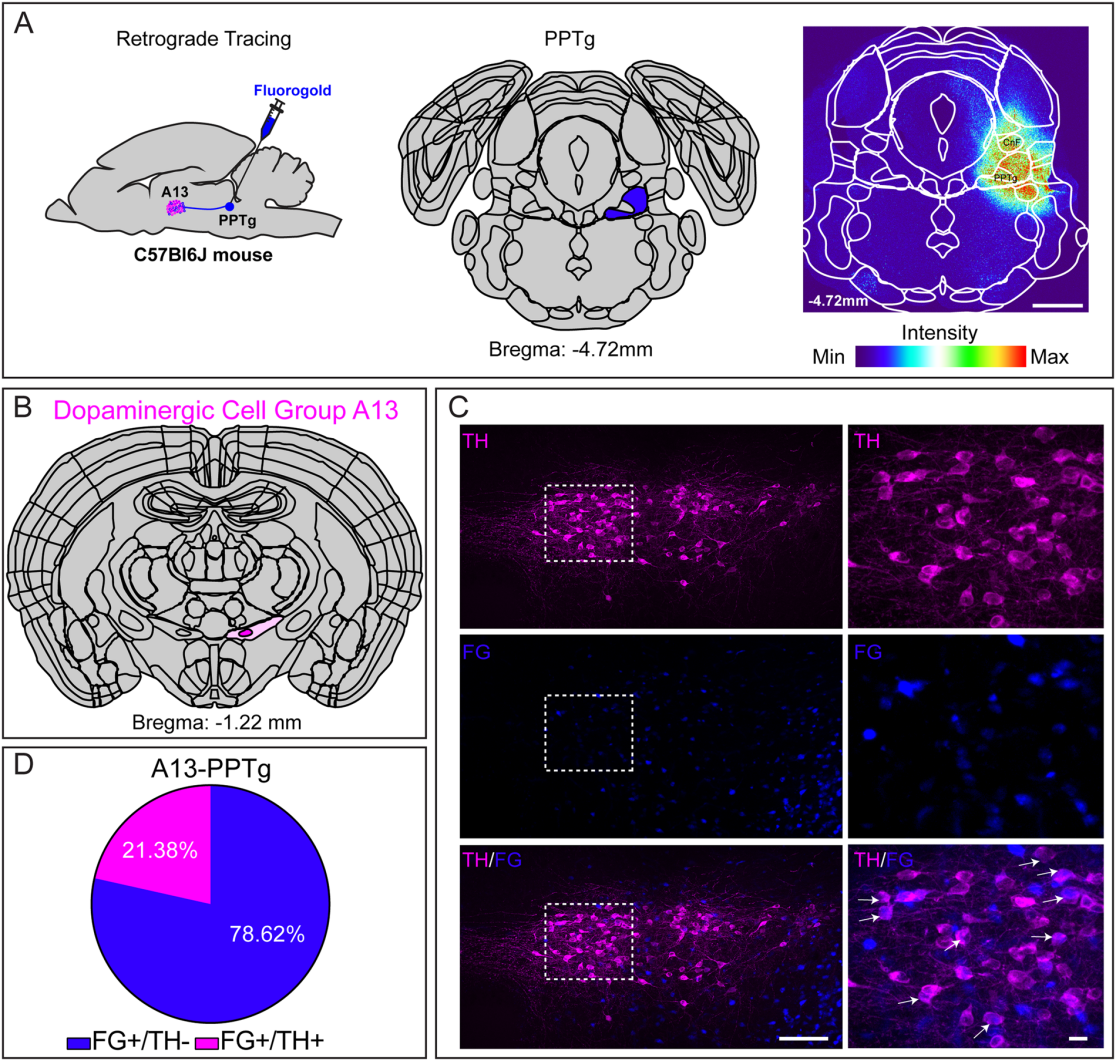
Dopaminergic A13 cells project to the PPTg. (**A**–**D**) A moderate number of the FG labelled cells (21.3%; n = 8 mice) co-expressed TH-ir. Scale bars A = 1000 µm, B = 100 µm and 20 µm. Atlas images adapted from template available from Allen Mouse Brain Atlas (2004)^39,90,91^.

To establish connectivity between the DAergic A13 neurons and the PPTg region of the ventral MLR, we injected a retrograde tracer FG into the PPTg region (Fig. 2A). The animals were included in the analysis if the core of injection was in the PPTg (Supplementary Figure 1). The spread from the core encroached onto neighbouring regions including MRN, LL, pontine reticular nucleus (PRN) and ventral portion of CnF. We observed several FG^+^ cell bodies in A13 (204.1 ± 23.42; n = 6 mice, Fig. 2B,C) in addition to the A11, ZI and LH (data not shown). We observed that 21.38% of total FG^+^ cells of A13 (43.63 ± 7.7 cells; n = 8 mice) were TH-ir (Fig. 2D) suggesting an important role for A13 neurons in the DAergic innervation of the PPTg.

#### Dorsal striatum

**Figure 3:**
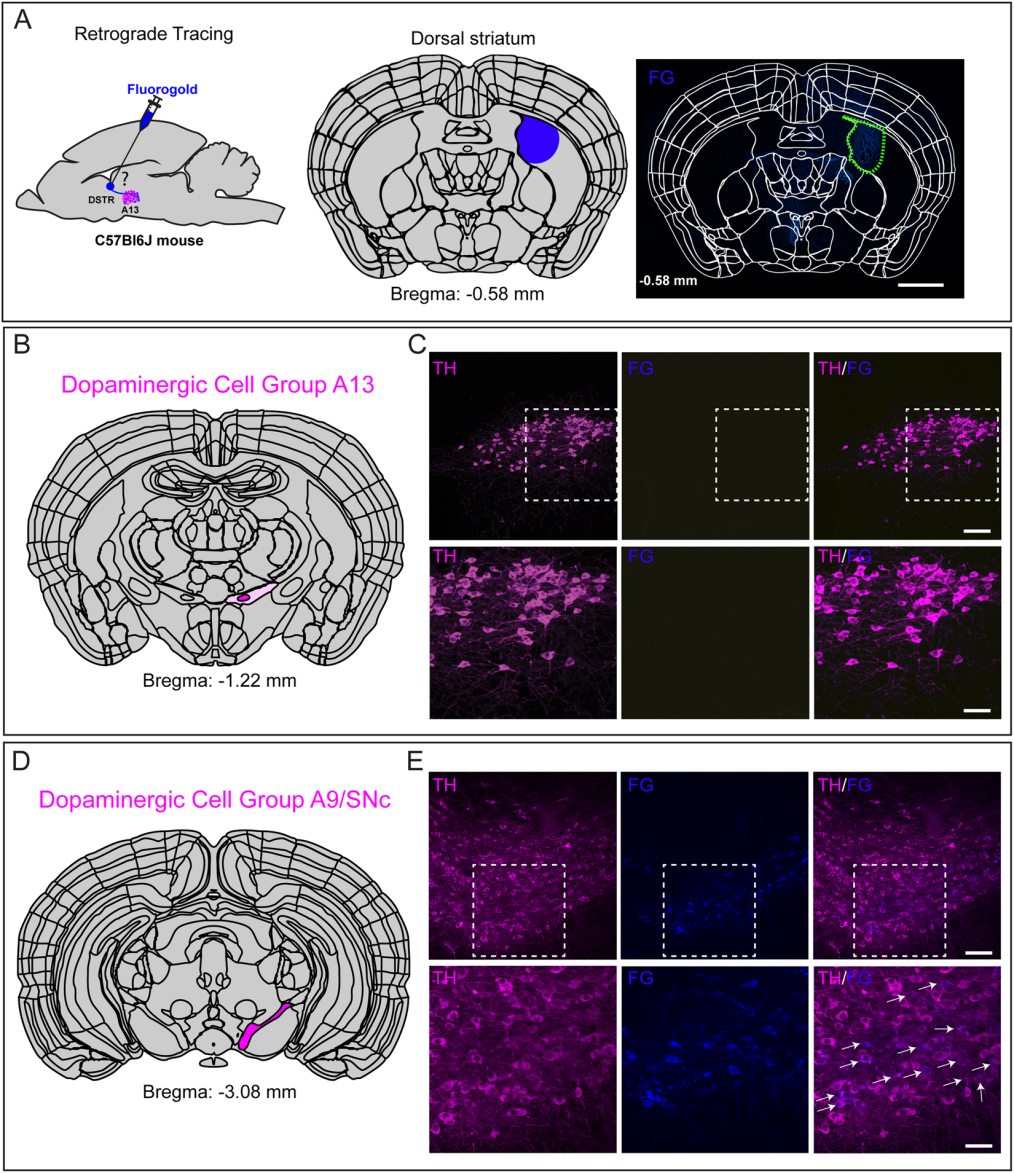
Lack of ascending dopaminergic A13 connectivity to the DSTR. (**A**–**C**) There was a lack of FG labelled cells in the A13 region which was identified by TH-ir (n = 9 mice). (**D**,**E**) Several FG^+^ cells with TH-ir were observed in SNc. Scale bars A = 1000 µm, B–E = 100 µm and 20 µm. Atlas images adapted from template available from Allen Mouse Brain Atlas (2004)^90,91^.

Next we examined whether DAergic A13 neurons have ascending projections to the DSTR in the brain. Mice were injected with FG in DSTR (Fig. 3A). To cover the rostrocaudal extent of DSTR, we injected over an AP bregma range from +1.00 mm to −0.50 mm. The core of FG injection and the spread was monitored in both the rostral and caudal directions from the injection site (bregma: +1.18 mm to −0.94 mm). The A13 TH-ir neurons did not show FG expression (n = 9 mice, Fig. 3B,C) but dense FG^+^/TH^+^ cell bodies were seen in the SNc/VTA region (Fig. 3D,E).

#### Gigantocellular reticular nucleus

**Figure 4:**
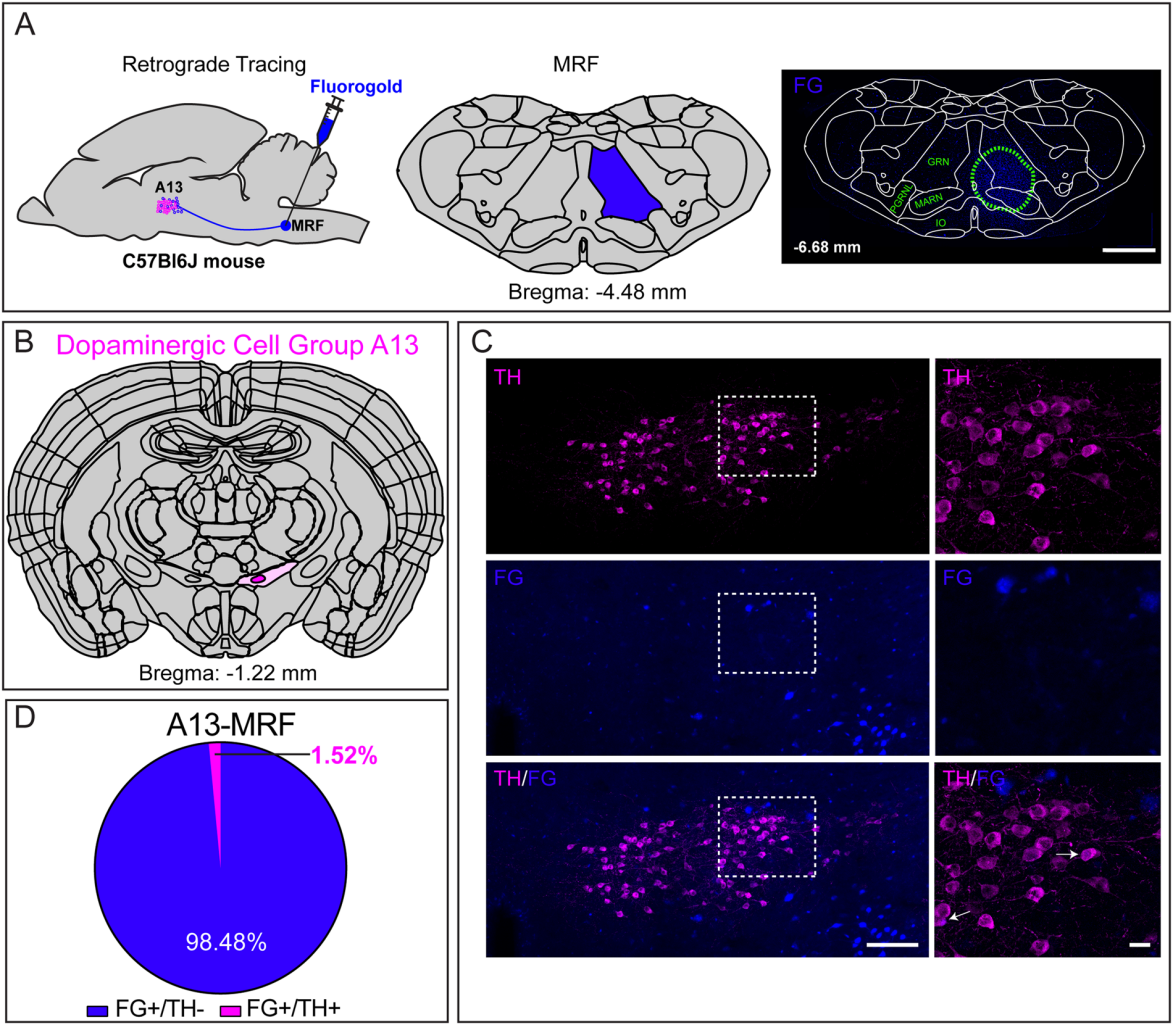
Dopaminergic A13 cells lack projections to the Gi in the MRF. (**A**–**D**) A few FG labelled cells with TH-ir were observed in the A13 region (1.5%, n = 6 mice). Scale bars A = 1000 µm, B,C = 100 µm and 20 µm. Atlas images adapted from template available from Allen Mouse Brain Atlas (2004)^39,90,91^.

To establish connectivity between DAergic A13 neurons and the MRF we injected a retrograde tracer FG into the Gi of the MRF (Fig. 4A). The Gi forms part of the MRF and is a hub for the control of spinal locomotor circuits^23^. The Gi is a large brain region so to cover its rostrocaudal extent, the animals were injected with FG in the Gi within a range of anterior-posterior (AP) bregma range from −6.20 mm to −6.68 mm. The animals were included in the analysis if the core of injection was in the Gi region with some spread to neighbouring regions including intermediate reticular nucleus (IRN), magnocellular reticular nucleus (MRN) and lateral part of paragigantocellular reticular nucleus (LPGi). We observed several FG^+^ cells in A13 (47.4 ± 13.4 cells; n = 6 mice, Fig. 4B,C) in addition to A11, ZI and LH in the vicinity (data not shown). We observed that 98.5% of total FG cells counted in A13 (46.7 ± 13.1 cells; n = 6 mice) were TH-ir negative; only rarely were TH-ir neurons co-labelled with FG observed (0.71 ± 0.4 cells; 1.5%; Fig. 4D). Regardless of the site of injection in the AP axis of Gi, most TH-ir neurons in A11 region were found to be positive for TH-ir (Whelan Lab, unpublished observations). This suggests that DAergic A13 is not a source of DAergic innervation to the Gi region of the MRF.

#### Lumbar spinal cord

**Figure 5:**
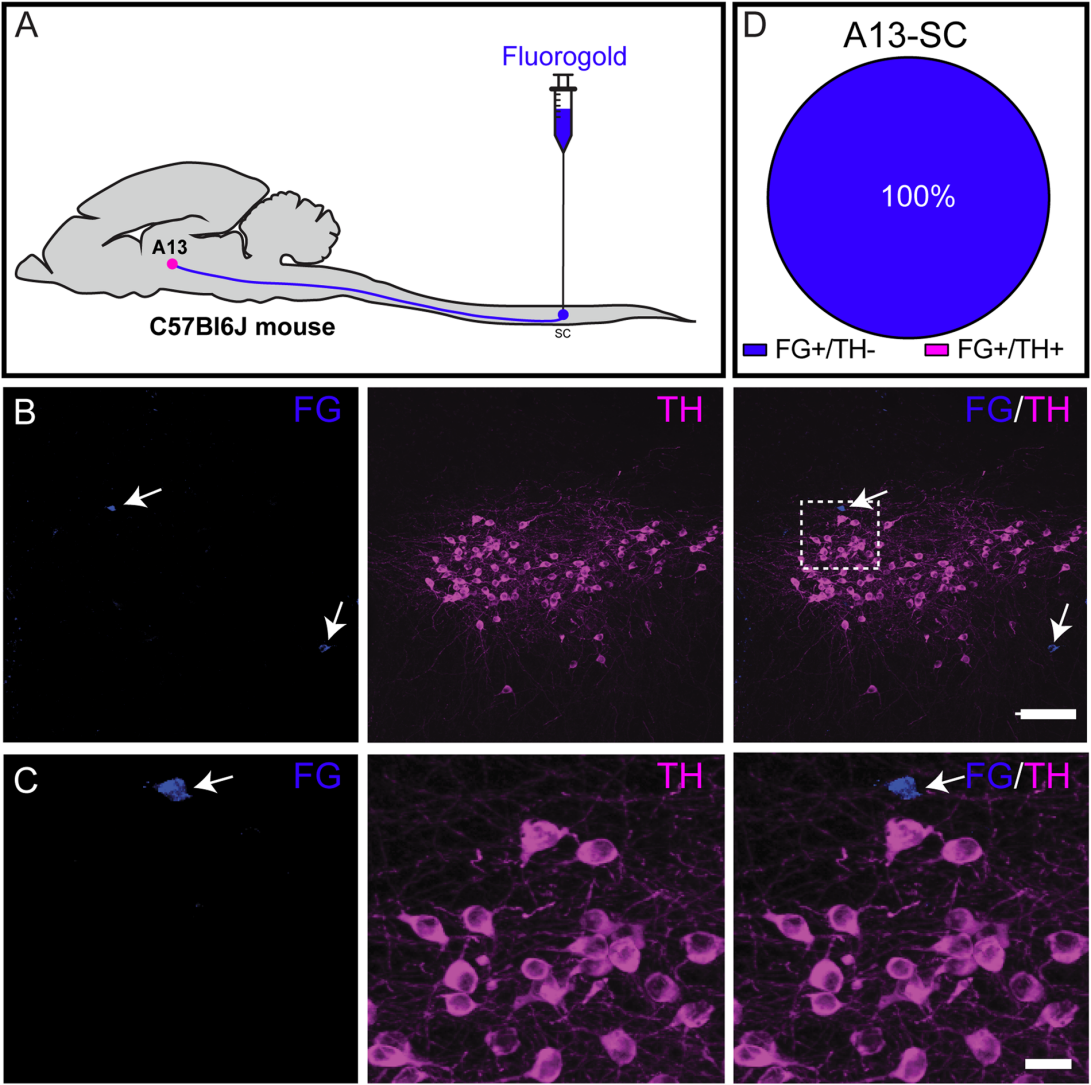
Retrograde tracing of spinally projecting dopaminergic A13 cells. (**A**–**D**) All the FG labelled cells in A13 region (100%; n = 6 mice) lack TH-ir. Scale bars B = 100 µm, C = 20 µm.

To examine projections from DAergic A13 neurons to the lumbar spinal cord, a retrograde tracer FG was injected into the lumbar (L2–L5) segments of the spinal cord of C57BL/6 mice (Fig. 5A). The FG injection spread was observed across lumbar segments (L1–L5) both in dorsal and ventral horns of the spinal cord. We observed FG^+^ cells in brainstem areas known to have projections to the lumbar spinal cord such as the locus coeruleus (LC) and the MRF (data not shown). We also found FG^+^ cells in the paraventricular hypothalamus (PVN) rostral to A13 cell group, validating our retrograde tracing paradigm from the lumbar spinal cord (data not shown). We reproduced our previously published observations showing FG^+^ cells in A11 also express TH-ir^15^ (data not shown). We observed FG^+^ cells in and around A13, ZI and lateral hypothalamus (LH). However, none of the FG labelled cells of A13 (10.33 ± 3.14 cells; n = 6 mice) were TH-ir suggesting that while neurons in the medial ZI project to the spinal cord they are not DAergic (Fig. 5B–D).

### DAergic A13 neurons have preferential connectivity pattern in the dorsal and ventral MLR

**Figure 6:**
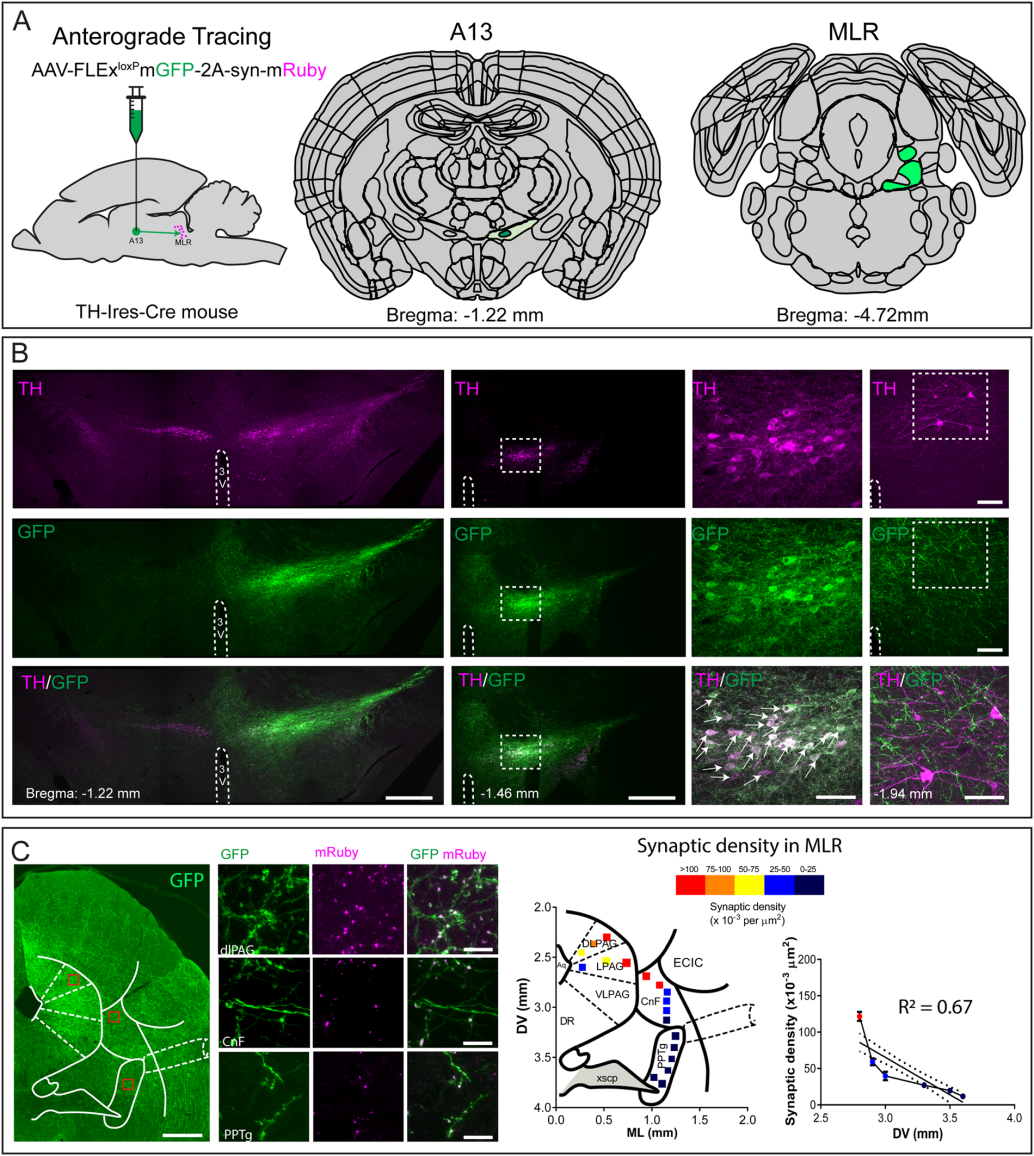
Anterograde tracing of dopaminergic A13 projection cells and synaptic puncta to the MLR. (**A**–**C**) Anterograde fibers (GFP^+^, green) were noticed with clear labeling of synaptic puncta (mRuby^+^, magenta) in dlPAG, CnF and PPTg. The density of synaptic puncta was high in dlPAG as expected. We observed a dorsoventral gradient in synaptic density with higher synaptic density of mRuby^+^ punctas in CnF showing a sharp decline in the PPTg region. Scale bars A = 1000 µm, B–D = 20 µm and 10 µm. Atlas images adapted from template available from Allen Mouse Brain Atlas (2004)^39,90,91^.

Our results using retrograde tracing indicate the presence of TH-ir projection cells with putative connectivity to both CnF and PPTg of the MLR. We noticed a higher number of FG^+^/TH-ir projection neurons in the A13 region in cases where the epicentre of FG injection was determined to be in CnF (68.83 ± 9.33 cells) compared to PPTg (43.63 ± 7.7 cells) indicating a preferential connectivity. However, this difference was not significant (p = 0.08, Mann-Whitney test). To further investigate the preferential connectivity of A13 TH-ir cells to CnF and PPTg of the MLR, we injected AAV-FLEx^loxp^-mGFP-2A-synaptophysin-mRuby in the A13 region of TH-IRES-Cre mice (Fig. 6A). While most GFP^+^ neurons were positive for TH-ir in the A13 region, we did observe few GFP^+^ neurons lacking TH-ir mainly in lateral division of zona incerta. We noticed that majority of the GFP^+^ neurons were TH-ir (82.82%, n = 3 mice, Fig. 6B) with a small proportion of GFP^+^ neurons (17.23%) lacking detectable levels of TH-ir. The TH-ir neurons of rostral A11 region did not show the presence of GFP confirming that GFP transfection was restricted to A13 region (Fig. 6B). Our results indicated the presence of GFP^+^ projection fibers (in green) and mRuby^+^ synaptic puncta (in magenta) in dorsolateral periaqueductal grey (dlPAG), one of the known target sites of DAergic A13 cells^31^ (Fig. 6C). We observed the presence of GFP^+^ fibres and mRuby^+^ synaptic puncta both in the CnF and PPTg (Figure C). We quantified synaptic puncta density in dlPAG, CnF and PPTg brain regions as described by Beier *et al*.^32^. Our results show that dlPAG has a high density of mRuby^+^ synaptic puncta confirming previous reports of A13 DAergic innervation of this region^31^ (Fig. 6C,D). We noticed higher density of these mRuby^+^ synaptic puncta in the CnF compared to the PPTg indicating a dorsoventral gradient of A13 connectivity to the MLR (Fig. 6C, n = 3 mice). We plotted slope of mRuby^+^ synaptic puncta against MLR dorsoventral coordinates. The density of mRuby^+^ synaptic puncta displayed a significant decline from CnF to the PPTg based on the dorsoventral coordinates (R^2^ = 0.67, F = 68.79, p < 0.0001, Fig. 6D) indicating preferential connectivity of DAergic A13 projections to the CnF region of the dorsal MLR compared with the PPTg of the ventral MLR.

### Morphological heterogeneity in the DAergic A13 cells

**Figure 7:**
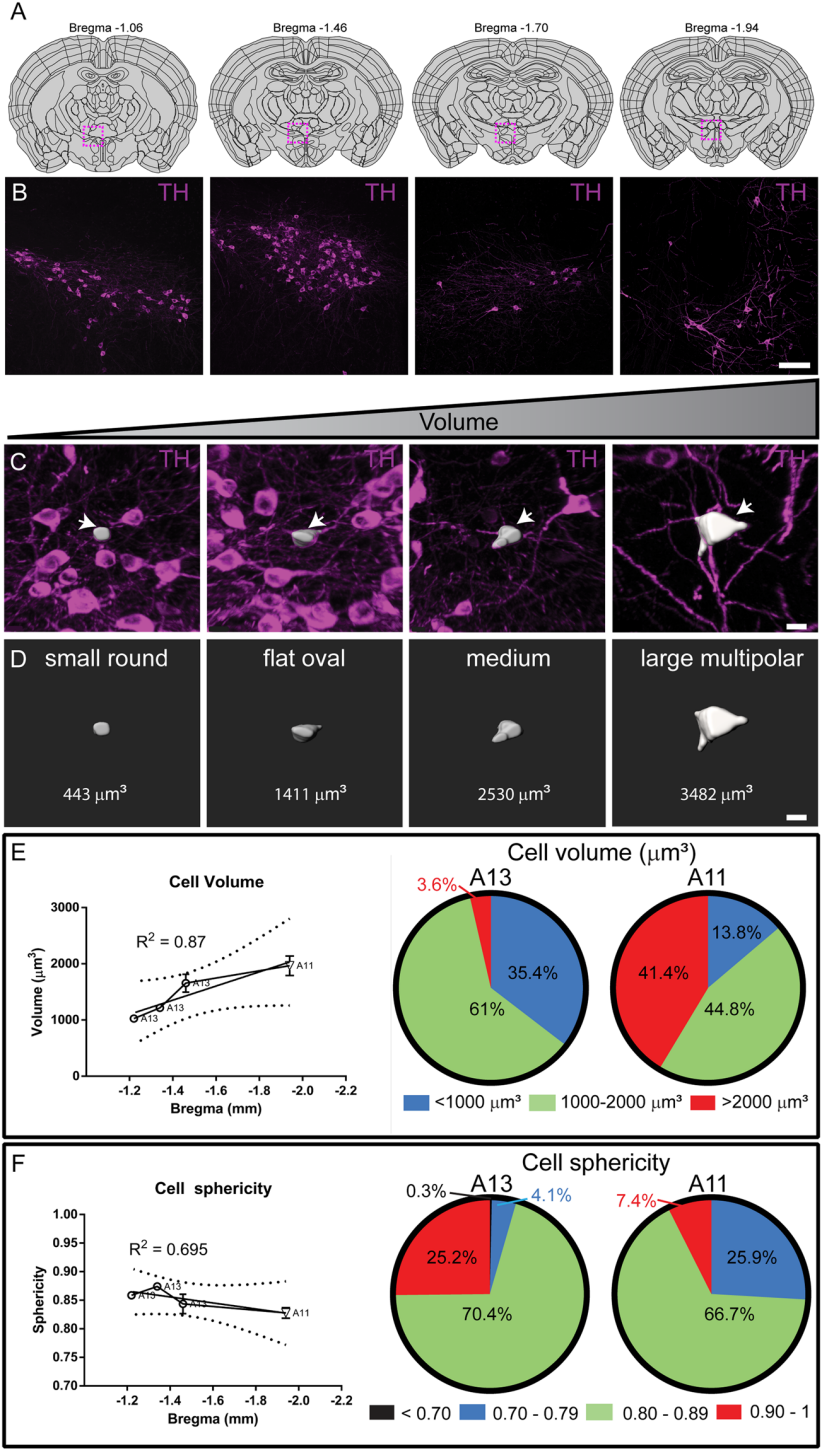
Heterogeneity in TH-ir cell morphology and size in A13. (**A**,**B**) TH-ir cells at rostral, medial and caudal A13 along with rostral A11. (**C–F**) The A13 region had dense clusters of TH-ir cells ranging from small round and oval to large pyramidal cells however the A11 region had a higher ratio of large TH-ir cells (>2000 µm^3^). The volume of cells from A13 to A11 region showed a rostral-caudal gradient with largest cells seen in A11 region. Scale bars B = 100 µm, C,D = 10 µm. Atlas images adapted from template available from Allen Mouse Brain Atlas (2004)^39,90,91^.

The DAergic A13 neurons have been previously reported to be present in a dense cluster in medial ZI and ventromedial to the mammillothalamic tract (Fig. 7A). Similarly, we observed that TH-ir cells were present in dense clusters in rostral and medial A13 regions with a decline in density and number of TH-ir cells in caudal A13 region (Fig. 7A,B). A large proportion of the TH-ir cells in the A13 region were small round and flat oval in appearance, but occasionally observed multipolar pyramidal cells as well (Fig. 7C,D). The average volume of TH-ir cells in A13 region was 1027 ± 348 µm^3^ in rostral, 1216 ± 444 µm^3^ in medial and 1675 ± 629 µm^3^ in caudal A13 region, whereas the average volume in rostral A11 region was 1966 ± 935 µm^3^. We observed that cell volume increases such that there is a rostrocaudal gradient from A13 to A11 (R^2^ = 0.85, F = 11.29, p = 0.78, Fig. 7E). We observed that the A13 comprised only 3.59% of TH-ir large volume cells (>2000 µm^3^) as compared to 41.38% of such large volume TH-ir cells in A11. In addition to the larger volume, A11 TH-ir cells were also distinct with multiple branched dendritic processes as compared to A13 TH-ir cells. We also observed a large population of TH-ir A13 cells (25.2%) comprised of small round cells with high sphericity (0.90-1), whereas a very small percentage of these cells (7.4%) were noted in A11 (Fig. 7F). We observed a decline following a rostrocaudal gradient from A13 to A11 region. (R^2^ = 0.66, F = 3.929, p = 0.18). These results point toward a cellular heterogeneity in DAergic A13 neurons.

### Non-canonical DAergic phenotype of the A13 cells

**Figure 8:**
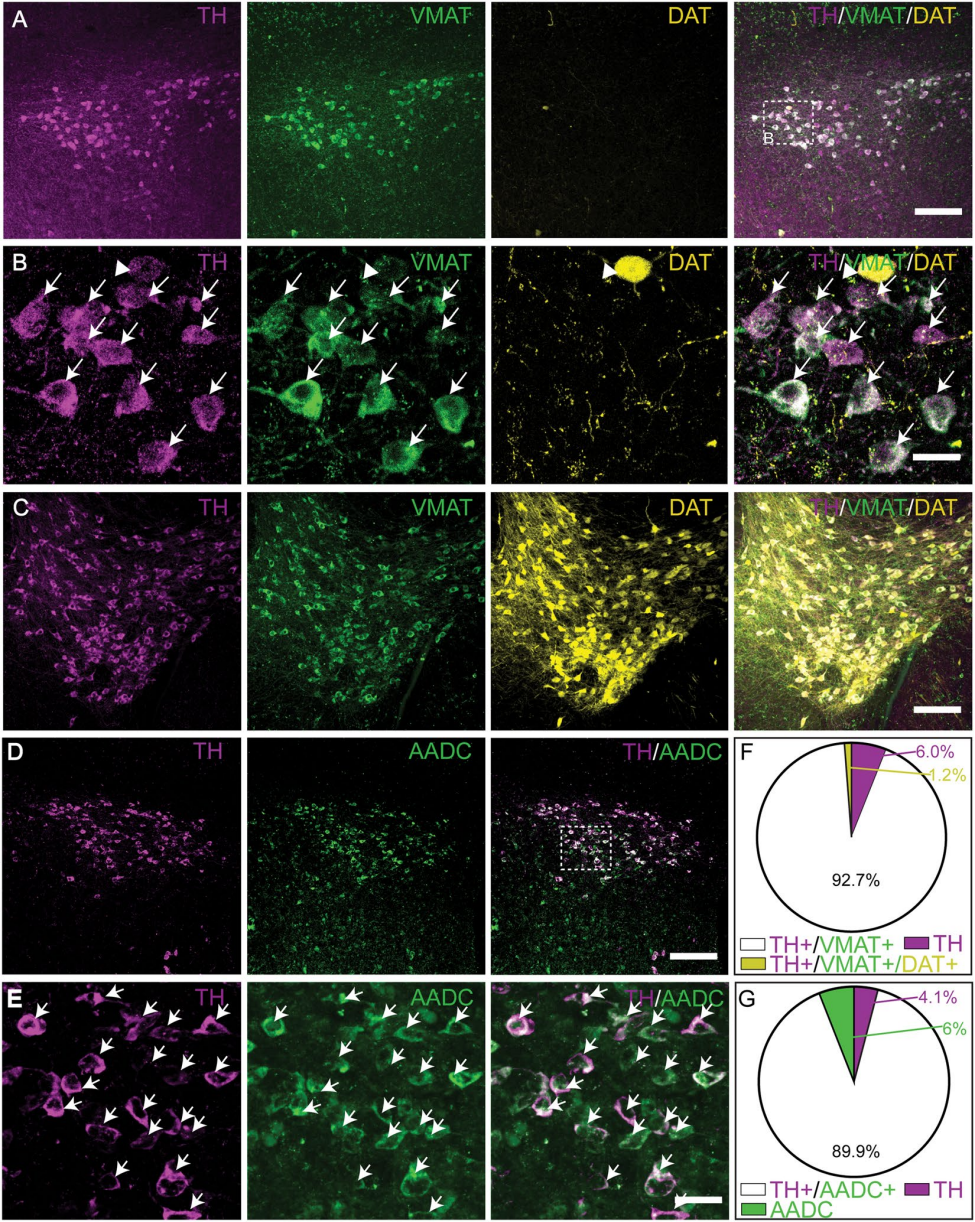
Dopaminergic phenotype of A13 cells. (**A**–**C**,**F**) Most TH-ir cells in A13 co-express VMAT2**-**ir and lack DAT expression; a few monoenzymatic cells with TH-ir were observed. (**D**,**E**,**G**) Most TH-ir cells in A13 co-express AADC-ir however a small percentage of monoenzymatic cells with either TH-ir or AADC-ir are seen. Scale bars A, C, D = 100 µm, B, E = 20 µm.

We next tested whether the A13 cells contain the full enzymatic complement present in the canonical DAergic neurons. We performed immunostaining to detect expression of AADC and TH in the A13 cells. The sections from the VTA were also processed as a positive control since this region contains AADC synthesizing DAergic neurons. As expected, AADC immunoreactive (AADC-ir) neurons in the VTA were mostly co-localized with TH-ir neurons (data not shown). In the A13, a large proportion of TH-ir neurons were co-labeled with AADC (89.9%), suggesting that these neurons contain the enzymatic machinery necessary to convert L-DOPA to DA (Fig. 8D, E, G). Interestingly, a small percentage of monoenzymatic cells exclusively expressing either TH (4.1%) or AADC (6%) were also observed^33^ (Fig. 8G). We next performed immunostaining to detect TH and VMAT2 in A13 cells of DAT-IRES-Cre-Ai14 reporter mice. In these mice, DAT-positive neurons are labelled with a tdTomato reporter construct. The sections from VTA were co-processed as a positive control since this region contains VMAT2 synthesizing and DAT-expressing DAergic neurons. The locus coeruleus (LC) contains VMAT2 expressing noradrenergic neurons but lacks DAT expression and provided a negative control for DAT expression. As expected, the VMAT2 expression in the VTA was intense and was co-localized with TH and the DAT-tdTomato reporter (Fig. 8C). LC noradrenergic neurons were VMAT2 immunoreactive (VMAT2-ir) and TH-ir, but displayed no DAT-tdTomato expression (data not shown). In the A13, a sizable proportion of TH-ir neurons co-expressed VMAT2 (92.7%, Fig. 8A,B,F), suggesting that these neurons contain the machinery necessary to package DA to vesicles. However, a small number of monoenzymatic cells were TH-ir but not VMAT2-ir (6%; Fig. 8F). In the A13, TH-ir neurons rarely showed DAT tdTomato expression (1.2%; Fig. 8A,B,F), suggesting that these neurons do not contain the machinery for DA reuptake. It is possible that NET, the noradrenergic transporter was expressed in A13, since reports from the neocortex show that NET can transport DA, albeit with lower affinities than DAT. We tested this possibility and found that while NET was expressed in the LC, it was not expressed in A13 (data not shown).

## Discussion

We show that the MLR receives DA connections from the A13 which lacks ascending DAergic projections to the DSTR. These projections are parallel to the canonical DAergic nigrostriatal motor pathway. Although non-DAergic neurons in the medial ZI were found to project to the spinal cord, we did not obtain evidence for a DAergic ZI descending projection. Collectively, these data suggest a refined medial ZI connectome onto locomotor regions of the brainstem and spinal cord.

The A13 cell group contains the necessary set of enzymes to synthesize DA (TH and AADC) in the mouse brain and package DA into vesicles (VMAT2). Initial characterization of monoamines in the mammalian brain reported A13 cells to contain DA in rat^34^, and later work using micro-spectrofluorimetric analysis confirmed the presence of DA and the absence of noradrenaline in rat A13 cells^35^. We observed that DAergic A13 neurons lack DAT expression similar to the A11^15^, the periventricular nucleus (A14), preoptic area (A15), and supraoptic nucleus^36^. Our finding of a lack of A13 NET expression is supported by data in mouse (Allen Brain Atlas; http://mouse.brain-map.org/) and the macaque monkey^37^. Work using DAT knockout mice shows that the mice are excitable, have increased DAergic tone, and exhibit pathologies typical of DA overproduction^38^. Therefore, it is reasonable to speculate that DA can be released from A13 projecting terminals even though they lack DAT. One possibility is that NET could transport the DA in A13. However, data from the Allen Brain Atlas shows the medial ZI to be negative for NET mRNA^39^, and our NET immunostaining revealed a lack of NET-ir in this region (data not shown). We observed morphologically distinct subpopulations of the A13 neurons that showed a rostrocaudal size gradient. Since smaller cells are generally more excitable, a hypothesis is that one would see more excitable cells in the rostral compared to the caudal A13.

Our work indicates that the A13 projects to areas of the brainstem associated with locomotor control, specifically the MLR. The MLR located on the mesopontine border is an established locomotor region in numerous species including lampreys, cats^1^, rats^3^, and mice^4^. Several attempts have been made to identify possible MLR sites in the brain of various species, but this issue remains contentious^3,40–43^. However, CnF in the dorsal MLR and PPTg in the ventral MLR are the two sites that have repeatedly been observed to induce locomotion^4,21,44–46^, and direct efferent projections to the lumbar spinal cord have been reported^45^. Moreover, DA was detected in the PPTg^47^ and CnF^48,49^ of the rat brain. Our data indicate DAergic A13 neurons have projections onto the PPTg. The PPTg can be characterized by heterogeneity in neuromodulatory types, including GABA, glutamate, acetylcholine, calcium-binding proteins and neuropeptides^50–55^. The PPTg has received considerable attention due to the dense innervations from not only the internal segment of the globus pallidus (GPi)^4^ and substantia nigra pars reticulata (SNr)^56,57^, but also direct DAergic input from SNc^5,7,10^. As such, alterations of this circuit are implicated in the symptoms of Parkinson’s disease (PD), including the freezing of gait. While the PPTg is associated with locomotion, it contributes to complex sensorimotor adaptions. An interesting possibility is that the DAergic A13 projections onto the PPTg may have functional implications in movement disorders.

Our data also show a projection from A13 to the CnF of the dorsal MLR, and interestingly stimulation of the CnF is reported to more effectively drive locomotion in both cats^58^, rats^59^, and mice^46^. The c-fos expression (marker of recent neural activity) after treadmill locomotion was detected more densely around CnF compared to PPTg in rats^60^. The CnF in mouse which lies within boundaries of presumptive MLR is reported to be a source of descending spinal projections^26,43,61^ and can effectively induce locomotion^4^. Although we optimized our retrograde FG injection protocol to localize injection core and spread in the target regions, we noticed some spread in the neighbouring regions. While we chose mice with the injection epicentre within targeted nuclei, we acknowledge that there will always be some spread to other sites. Our results of anterograde tracing reveal that DAergic A13 neurons project to both CnF and PPTg, and the existence of a dorsoventral gradient in mRuby^+^ synaptic puncta suggesting preferential connectivity to the CnF and PPTg that may have functional implications. That said, its projections to CnF and PPTg could act synergistically, or as redundant, parallel pathways to modulate downstream motor centres. In addition to the PPTg receiving basal ganglia outputs and a direct DAergic innervation from SNc, our findings illustrate the A13 as an additional source of DA to the MLR.

While our work shows projections onto both the PPTg and CnF, we don’t show specific projections onto classes of neurons. Roseberry and colleagues^4^ found that activity of the glutamatergic cells within the MLR correlates with spontaneous locomotor episodes and is sufficient to drive locomotor bouts. In contrast to MLR glutamatergic cells, GABAergic cells are associated with deceleration and stopping^4^. This suggests that GABAergic and glutamatergic cells within the MLR may synergistically control braking and accelerate locomotor behaviors. The activation of cholinergic cells in MLR alone is not sufficient to elicit a locomotor bout but instead results in acceleration of locomotion during an episode, which is in line with previous reports in the lampreys^62^. The morphological heterogeneity observed in A13 DAergic cells may have a functional perspective, and indeed it could be hypothesized that they may project differentially onto classes of neurons within the MLR, with braking being due to activation of small, easily excited cells, and gait being activated by cells requiring higher levels of excitability.

Our work indicates a lack of ascending connectivity of A13 DAergic cells to the DSTR. A similar lack of ascending connectivity from A13 DAergic neurons to the nucleus accumbens has been reported previously^63^, which combined with our present results indicates a lack of ascending striatal connectivity of A13 DAergic cells. However, this does not rule out any possible nigrostriatal outputs to A13 DAergic cells. Our results point to a novel, distinct and parallel dopaminergic innervation onto PPTg and CnF regions from the A13 region. The DAergic A13 cells also project to the dorsolateral periaqueductal grey^31^ implicated in defensive and panic behaviors and to the medial superior colliculus^64^ implicated in attention, orientation and aversive behaviors. The A13 cell group projects to the central nucleus of the amygdala^63^, involved in the expression of fear and other emotional behaviors^65^. The thalamus in primate brain has been reported to receive dopaminergic projections from A13^37^; however, the potential role of this projection in sensorimotor integration has yet to be studied. Also, the paraventricular nucleus of thalamus (PVT) of rat receives dopaminergic inputs from ventrorostral A10 (excluding VTA), A11, A13, A15 and midbrain dorsocaudal A10 within the periaqueductal gray^66^. Interestingly, the PVT has been reported to gate and process the signals relevant to reward and danger, which can subsequently converge on pertinent circuits for approach or avoidance, respectively^67^.

Our work shows that the DAergic A13 neurons lack substantial projections onto the Gi of MRF. The MRF contains groups of diffusely located nuclei that form an important integration point for the control of locomotion and contain neurons that project onto interneurons and motoneurons of the cervical and lumbar spinal cord^28,68^. The reticulospinal cells in MRF descend ipsilaterally via the ventrolateral and ventromedial funiculi^28,29^ but contralateral projections have also been reported^69–71^. Interestingly, work focused on stimulation of ventrolateral tracts show that they can activate the spinal central pattern generators (CPGs), and acute lesions of the ventrolateral tract result in changes in the gait of freely moving animals^72^. Our results indicate DAergic A13 neurons lack descending connectivity to the Gi region of MRF. However, the possible connectivity to other nuclei in MRF cannot be excluded.

Our data shows a lack of connectivity between DAergic A13 cells and the lumbar spinal cord, an area encompassing the spinal locomotor generating region which is sensitive to DA neuromodulation^60,73–76^. This contrasts with the adjacent A11 which shows DAergic projections to the SC. Our observation of non-DAergic projections from the medial ZI to the spinal cord agrees with work from others^77^ using mice^14^, rats^78,79^ and primates^80^, although one study in rabbits found A13 DAergic projections^81^. An important difference is that mouse and rat work was performed using a retrograde labelling of A13 to lumbar segments whereas work in the rabbit focused on the A13 projections to cervical segments. On the other hand, injections in the cervical segments of the monkey with FG did not show TH-ir^+^ projecting A13 neurons^80^. Future studies are needed to explore neurochemical phenotypes of non-TH-ir^+^ labelled FG neurons projecting to the lumbar SC.

**Figure 9:**
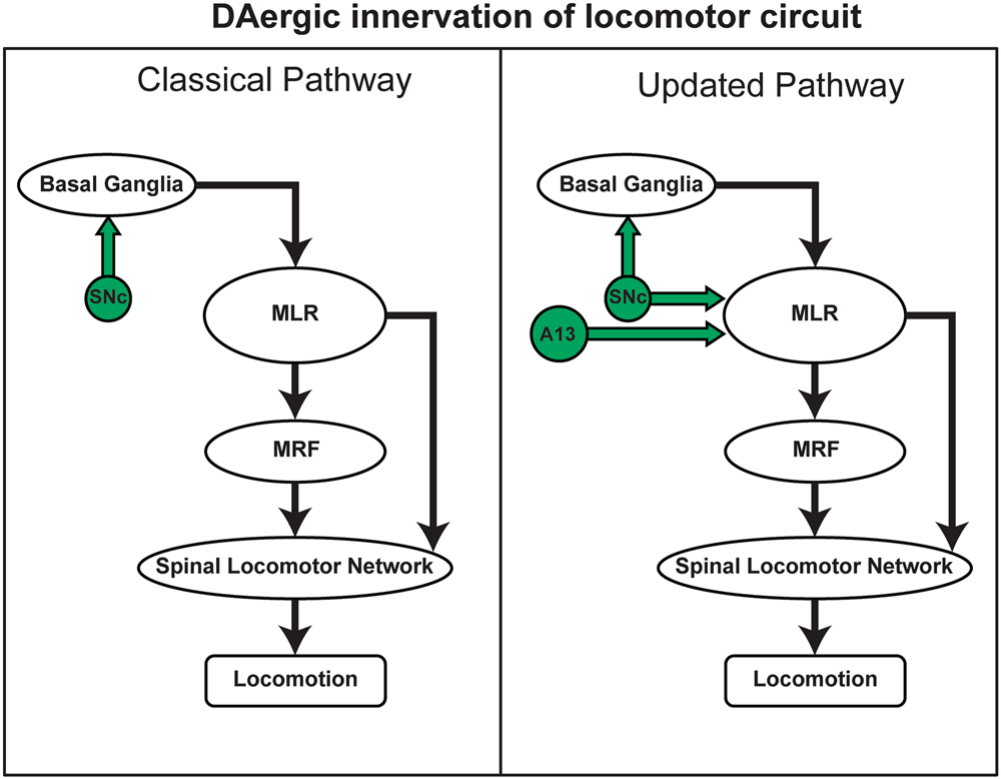
Dopaminergic innervations of the locomotor centers: The schematic shows connectivity between basal ganglia, MLR, MRF and spinal locomotor network that is known to produce locomotion. The DAergic contribution to the classical pathway consisted ascending DAergic projections from SNc to basal ganglia. However findings from our present work and others^7^ show novel parallel descending DAergic projections from A13 and SNc to MLR respectively.

A significant aspect of our work is the discovery of DAergic A13 as source of descending dopaminergic innervations of MLR in addition to SNc^7^. This pathway appears to be anatomically distinct to the nigrostriatal pathway and sheds new light on the complexity of the DAergic connectome. The function of the identified pathway is unclear, but its projections suggest that it contributes to motor control (Fig. 9). Indeed, electrical stimulation of the medial ZI where the A13 resides in the mouse is associated with locomotor and postural activity^21,82–86^. The specific role of these descending A13 DAergic projections to MLR is unknown, but they may contribute to the reward-based selection of motor programs as observed in other areas of the brain^87–89^. The existence of this novel descending DAergic innervations to MLR may have implications for our understanding of the role of DA in motor control under physiological and pathological conditions.

## Materials and Methods

### Ethics statement

All animal experiments were approved by the University of Calgary Health Sciences Animal Care Committee (Protocol: AC15-0016), in accordance with the Canadian Council for Animal Care.

### Animals

Adult male 8–16 week old C57BL/6 mice (Charles River Laboratories, Senneville, Quebec, Canada), TH-IRES-Cre knock-in mice (gift from Dr. Antoine Adamantidis, McGill University, Canada –source; EM: 00254;B6.129 × 1-Thtm1(cre)Te/Kieg; European Mouse Mutant Archive), DAT-IRES-Cre knock-in mice (Jackson Labs, Bar Harbor, Maine, USA; B6.SJL-Slc6a3tm1.1(cre)Bkmn/J), ROSA26 tdTomato reporter mice (Jackson labs; B6.Cg-Gt(ROSA)26Sortm14(CAG-TdTomato)Hze/J (Ai14)) were used in the study. We then crossed the lines to obtain TH-IRES-Cre homozygous, DAT-IRES-Cre-Ai14 heterozygous mice. Pairs of homozygous DAT-IRES-Cre (female) or Ai14 (ROSA26tdTomato; male) genotypes were mated, and the resulting heterozygous DAT-IRES-Cre-Ai14 male offspring were used in subsequent experiments. All mice were genotyped using DNA extracted from ear notches using the Kapa mouse genotyping kit (Kapa Biosystems, Roche Canada, Mississauga, Ontario, Canada) according to manufacturer’s instructions. Genotyping of the TH-IRES-Cre, DAT-IRES-Cre, reporter Ai14 and DAT-IRES-Cre-Ai14 mice were performed in a similar manner, using the primers recommended by the supplier (Jackson Labs, Bar Harbor, Maine, USA). All mice were housed on a 12:12 hour light: dark schedule (lights on at 07:00 – off at 19:00) with ad libitum access to food and water.

### Tracer Injections

For tracing connectivity of A13 DAergic cells to the lumbar spinal cord, male C57BL/6 mice (n = 6) were anesthetized with isoflurane (1.5%) and a partial laminectomy was performed between vertebral segments T13-L1 to expose the middle of the lumbar enlargement of the spinal cord. The dura was carefully cut with a 34-gauge needle run perpendicular to the spinal cord along the dura, until cerebrospinal fluid was expelled from beneath the dura. The dura was further resected by using fine 45-5 forceps (Fine Science Tools, cat. no. 11251-35) to grasp the tissue and finally straight spring scissors (Fine Science Tools, cat. no. 15000-08) to enlarge the hole. After removal of the dura to expose the dorsal surface of the SC, the fluorescent tracer Fluoro-Gold (FG; 2% w/v in Saline; Fluorochrome) was pressure injected through a glass capillary pulled to a fine tip (Drummond Scientific, PA, USA; Puller Narishige, diameter 15–20 mm) and Nanoject II apparatus (Drummond Scientific, PA, USA). For each injection track, each mouse received 30 nl delivered both dorsally and ventrally. To ensure adequate coverage, each mouse received 4 injections per side of the SC (L2 to L5) for a total of 8 injections bilaterally. The muscles and skin were sutured, and the mice were given buprenorphine (0.1 mg/kg) for post-surgery analgesia. The animals were then returned to the animal facility for at least three weeks before they were sacrificed, and FG injection sites were verified.

To investigate descending connectivity from A13 to brainstem locomotor regions (MLR and MRF), 8–12 week old C57BL/6 mice were injected with 2% FG at cuneiform nucleus (CnF): AP −4.5 mm; ML −1.10 mm to −1.25 mm from Bregma; DV −2.90 mm to −3.1 mm from the dura; pedunculopontine tegmental nucleus (PPTg): AP −4.36 mm to −4.60 mm; ML −1.1 mm to −1.2 mm from bregma; DV −3.5 mm to −3.7 mm from the dura; MRF: AP −6.20 mm to −6.62 mm; ML −0.5 mm to 0.6 mm from bregma; DV −4.7 mm from the dura) unilaterally (total volume of 210 nl; n = 6–8/region). In a separate experiment to confirm the presence of anterograde projection fibers with synaptic puncta, 8–12 week old TH-IRES-Cre mice received unilateral injections of AAV-FLEx^loxp^-mGFP-T2A-synaptophysin-mRuby^32^ into the A13 (AP −1.22 mm; ML −0.5 mm from the Bregma; DV −4.5 mm from the dura, n = 3 mice). Animals were sacrificed three weeks after surgery and FG injection sites were verified.

To investigate ascending connectivity from A13 DAergic cells to dorsal striatum (DSTR), 8–12-week-old TH-IRES-Cre mice (n = 9 mice) received bilateral pressure injections of 2% FG in DSTR (AP +1.00 mm to −0.50; ML −1.75 mm to −2.50 mm from the Bregma; DV −2.25 mm to −3.00 mm from the dura) in a total volume of 198 nl (11 injections of 18 nl).

### Immunohistochemistry

Mice were deeply anesthetized with isoflurane (2%) and transcardially perfused with phosphate-buffered saline (PBS), followed by 10% formalin in PBS. Brains and spinal cords were placed in 10% formalin for up to 4 hours followed by 30% sucrose (w/v in PBS) for cryoprotection. 40 µM coronal brain sections, as well as 20 µM transverse and sagittal spinal cord sections were obtained using a cryostat (Leica CM1850 UV, Leica Biosystems, Ontario, Canada). The sliced brain and spinal cord sections were collected in a staggered fashion and placed into three and four consecutive wells, respectively. Spinal cord slices were mounted across 4 slides in a staggered fashion and warmed on a hot plate (40^o^ C) before washing. Rinses were performed before and between incubations with 0.1 M PBS before and between incubations, followed by one 20 minute wash in PBS with 0.5% Triton-X 100 (Sigma-Aldrich, St. Louis, MO, USA). Sections were incubated in blocking solution (5% donkey and 5% goat serum in PBS with 0.1%Triton-X 100) for 1 hour and blocking solution was used in subsequent antibody incubations. The details of primary antibodies used are provided in Table 1. Controls were included where the primary antibody was omitted to check for non-specific binding of the secondary antibodies. Free-floating brain sections were then mounted onto Superfrost^TM^ slides, coated with Vectashield^TM^ (H-1000, Vector, Burlingame, CA, USA) and cover-slipped. All primary and secondary antibodies, along with their conjugates, are presented in Table 1. Fluorescent images were collected using the following microscopes; Nikon Eclipse C1si spectral confocal microscope, Nikon A1R MP^+^ running in confocal mode and Olympus BX51 epifluorescence microscope. The objectives used were 4 × (NA 0.13), 20 × Plan Apo DIC (NA 0.75), 20 × Plan Fluor (NA 0.75), 60 × Plan Apo IR (NA 1.27). The lasers used were centred on 405 nm (with 450/50 emission filter), 488 nm (with 515/30 emission filter), and 561 nm (with a 590/50 emission filter) wavelengths. The 20 × images were taken with z-step 0.5–1 µm, and the 60 × with z-step 0.15 µm. Stacked images were acquired by averaging 2–4 frames with a resolution of 1024 × 1024 or 512 × 512. Offline image processing included maximal intensity projections conducted using NIS-Elements Advanced Research Version 4.10 (Nikon Canada Inc., Mississauga, Ontario, Canada). To quantify cell morphology features such as volume and sphericity, TH immunostained sections from A13 and A11 regions were imaged as described above. Surfaces were created for each cell body using Imaris 8.4 software (Bitplane AG, Zurich, Switzerland) and volume (µm^3^) and sphericity (0 to 1) values were measured. Data is represented as mean ± SEM.

**Table 1:** Primary Antibodies.

Antigen	Lab code	Donor species	Dilution/incubation time	Commercial Source
TH	AB112	Rabbit	1:1000 Overnight at room temperature	Abcam Inc., Toronto, ON, Canada
TH	AB1542	Sheep	1:500 Overnight at room temperature	EMD Millipore, Billercia, MA, USA
AADC	NBP1-56918	Rabbit	1:500 60 hours at 4 °C	Novus Biologicals, Littleton, CO, USA
VMAT2	H-V008	Rabbit	1:500 60 hours at 4 °C	Phoenix Pharmaceuticals, Burlingame, CA, USA
FG	NM-101	Guinea pig	1:500 Overnight at room temperature	Protos Biotech Corp, New York, NY, USA
GFP	GFP-1020	Chicken	1:1000 Overnight at room temperature	Aves Laboratories, Tigard, OR, USA
mCherry	CPCA-mCherry	Chicken	1: 2000 Overnight at room temperature	Encor Biotech Inc., Gainesville, FL, USA

### Synaptic puncta quantification

Methods for synaptic puncta quantification were adapted from Beier *et al*.^32^. To quantify the density of mRuby-labelled puncta from A13 TH-ir neuron termini in the CnF and PPTg, 100 nl of *AAV-FLEx*^*loxP*^*-mGFP-2A-synaptophysin-mRuby* was injected into the A13 of *TH-IRES-Cre* mice (n = 3), and sections were cut at a thickness of 40 μm. Floating sections were stained using anti-mCherry and anti-GFP antibodies (Table 1). Sections were imaged on a Leica SP8 confocal microscope using a 63 × objective, with image stacks at 0.44 μm intervals using 2 × averaging and 2 × optical zoom. Three images were taken of puncta in the dorsolateral periaqueductal grey (dlPAG), CnF and PPTg of each brain. Images were analysed using Imaris (Bitplane). The spots function was used to estimate the number of mRuby^+^ puncta. Data from the three slices from dlPAG, CnF and PPTg were averaged for each brain.

### Statistical Analysis

Statistical analyses were performed in GraphPad Prism 6. A non-parametric Mann-Whitney test was conducted comparing between two independent groups (i.e. CnF vs. PPTg). For synaptic density, cell volume and sphericity a regression analysis was performed. Data are reported as mean ± standard error mean (SEM) and P values less than 0.05 were considered significant.

## Electronic supplementary material

Supplementary Fig. 1
