# Supplementary material for: Parallel descending dopaminergic connectivity of A13 cells to the brainstem locomotor centers
Source: Sci Rep. 2018 May 22;8:7972. doi: 10.1038/s41598-018-25908-5 (PMC5964077; doi:10.1038/s41598-018-25908-5)
Supplement: Supplementary file 1 — Supplementary Fig. 1 [file 41598_2018_25908_MOESM1_ESM.pdf]

Parallel descending dopaminergic connectivity of A13 cells to the brainstem locomotor centers.

Sandeep Sharma, Linda H. Kim, Kyle A. Mayr, David A. Elliott, Patrick J. Whelan

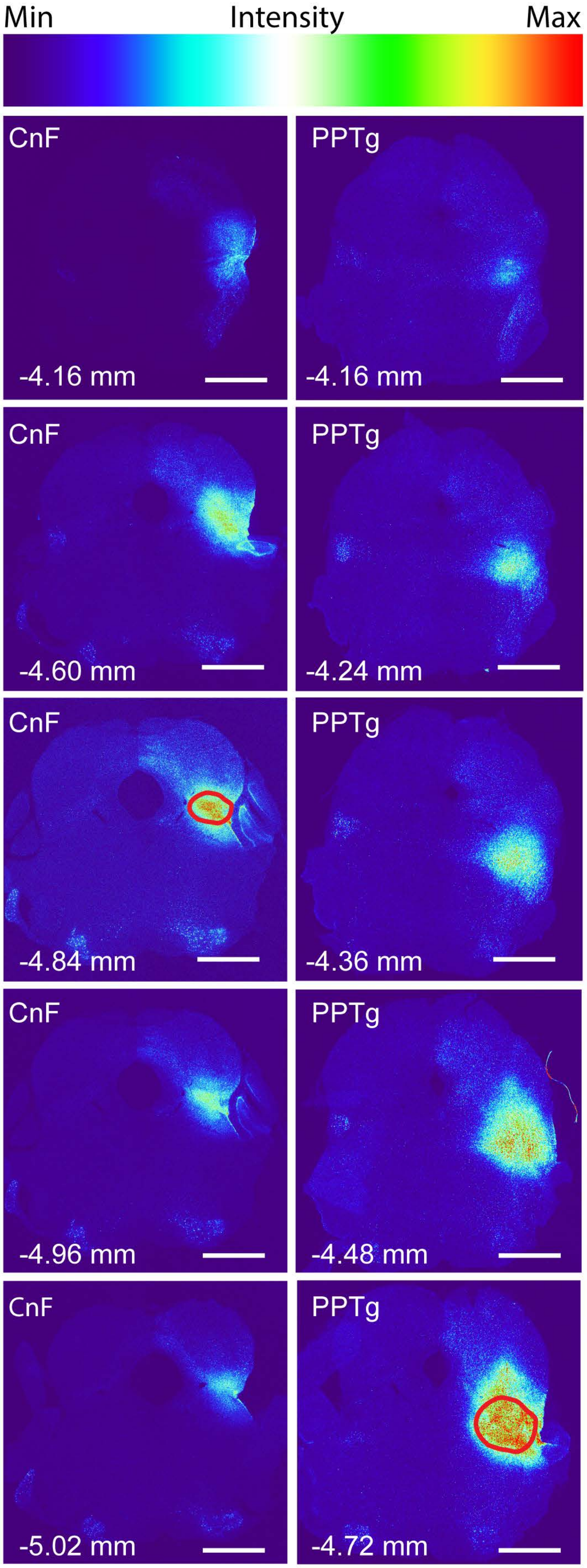
Min
Intensity
Max
CnF
-4.16 mm
PPTg
-4.16 mm
CnF
-4.60 mm
PPTg
-4.24 mm
CnF
-4.84 mm
PPTg
-4.36 mm
CnF
-4.96 mm
PPTg
-4.48 mm
CnF
-5.02 mm
PPTg
-4.72 mm
